# Glycoprotein N of Human Cytomegalovirus Protects the Virus from Neutralizing Antibodies

**DOI:** 10.1371/journal.ppat.1002999

**Published:** 2012-10-25

**Authors:** Barbara Kropff, Christiane Burkhardt, Juliane Schott, Jens Nentwich, Tanja Fisch, William Britt, Michael Mach

**Affiliations:** 1 Institut für Klinische und Molekulare Virologie, Friedrich-Alexander-Universität, Erlangen-Nürnberg, Germany; 2 Department of Pediatrics, University of Alabama Birmingham, Birmingham, Alabama, United States of America; Oregon Health and Science University, United States of America

## Abstract

Herpes viruses persist in the infected host and are transmitted between hosts in the presence of a fully functional humoral immune response, suggesting that they can evade neutralization by antiviral antibodies. Human cytomegalovirus (HCMV) encodes a number of polymorphic highly glycosylated virion glycoproteins (g), including the essential envelope glycoprotein, gN. We have tested the hypothesis that glycosylation of gN contributes to resistance of the virus to neutralizing antibodies. Recombinant viruses carrying deletions in serine/threonine rich sequences within the glycosylated surface domain of gN were constructed in the genetic background of HCMV strain AD169. The deletions had no influence on the formation of the gM/gN complex and *in vitro* replication of the respective viruses compared to the parent virus. The gN-truncated viruses were significantly more susceptible to neutralization by a gN-specific monoclonal antibody and in addition by a number of gB- and gH-specific monoclonal antibodies. Sera from individuals previously infected with HCMV also more efficiently neutralized gN-truncated viruses. Immunization of mice with viruses that expressed the truncated forms of gN resulted in significantly higher serum neutralizing antibody titers against the homologous strain that was accompanied by increased antibody titers against known neutralizing epitopes on gB and gH. Importantly, neutralization activity of sera from animals immunized with gN-truncated virus did not exhibit enhanced neutralizing activity against the parental wild type virus carrying the fully glycosylated wild type gN. Our results indicate that the extensive glycosylation of gN could represent a potentially important mechanism by which HCMV neutralization by a number of different antibody reactivities can be inhibited.

## Author Summary

Herpes viruses are transmitted between individuals in cell free form and successful spread benefits from mechanisms that limit the loss of infectivity by the activity of virus neutralizing antibodies. Human cytomegalovirus (HCMV) is an important pathogen and understanding how the virus can evade antiviral antibodies may be clinically relevant. HCMV particles contain a number of highly polymorphic, extensively glycosylated envelope proteins, one of which is glycoprotein N (gN). This protein is essential for replication of HCMV. We have hypothesized that the extensive glycosylation of gN may serve as a tool to evade neutralization by antiviral antibodies. Recombinant viruses were generated expressing gN proteins with reduced glycan modification. The loss of glycan modification had no detectable influence on the *in vitro* replication of the respective viruses. However, the recombinant viruses containing under-glycosylated forms of gN were significantly more susceptible to neutralization by a diverse array of antibody reactivities. Immunization of mice with viruses carrying fewer glycan modification induced significantly higher antibody titers against the homologous virus; however, the neutralization titers against the fully glycosylated virions, were not enhanced. Our results indicate that glycosylation of gN of HCMV represents a potentially important mechanism for evasion of antibody-mediated neutralization by a number of different antibody specificities.

## Introduction

Cytomegaloviruses (CMV) have co-evolved with their respective hosts. During this long and continuing co-evolution these viruses have adapted to the host defense systems and vice versa to allow the life-long persistence of these viruses. As a result, infections in immunocompetent hosts are generally asymptomatic and a life-long persistent/latent infection is readily established. Development of symptoms or disease is prevented by a multilayered, in large parts redundant, innate as well as adaptive immune response [1]. Persistence and transmission between hosts eventually requires the evasion of immune control. Multiple mechanisms that permit evasion of immune control by the innate and adaptive cellular immune responses have been extensively documented [1]–[3]. In contrast, very little is known about mechanisms by which CMV can evade humoral immune responses that presumably consist of antiviral antibodies that potentially neutralize free virus or destroy infected cells via antibody mediated cytotoxicity. Since viral transmission between hosts in a community setting is thought to occur via cell free virus in most cases that have been studied, evading virus neutralizing antibodies is essential for successful spread and persistence of CMVs in the population.

On the population level, the extensive strain polymorphism that has been documented in different human and animal CMVs could serve as an immune evasion strategy [4]–[6]. CMV strains exhibiting antigenic and genetic variability are capable of super-infecting immune hosts and can be readily transmitted between immune individuals [7], [8]. Transmission of super-infecting strains has been well documented during pregnancy or following organ transplantation [9], [10]. Strain-specific virus neutralization is potentially a contributing factor to this phenomenon and strain-specific neutralization has been observed in a number of studies [11], [12]. Thus, virus strain-polymorphism can be considered as a mechanism that permits successful maintenance of CMV within the host population.

To evade neutralizing antibodies on the level of an individual viral strain within a single host requires an evasion strategy other than virus strain-polymorphism. The development of virus mutants during virus persistence in an individual host that resist virus neutralizing antibodies is, based on existing data, only a theoretical possibility. Herpes viruses in general are believed to be genetically stable secondary to the proofreading activity of their DNA polymerase. Moreover, the neutralization of CMV *in vivo* almost certainly involves antibodies directed at a multitude of different viral antigens. Thus, multiple mutations would be required for evasion of virus neutralizing activities that are likely to be present in a single individual host. Induction of neutralizing and non-neutralizing antibodies which compete for binding to the same antigenic determinant has been described as one possible mechanism for evasion of neutralization but this has been shown only for a single antigenic site on glycoprotein (g) B [13], [14]. Another possibility for viral evasion of neutralizing antibodies is the addition of carbohydrate to virion envelope glycoproteins that can alter antibody binding, a mechanism that has been extensively documented for viruses such as HIV and influenza, among others [15].

Human CMV (HCMV) is a structurally complex virus which contains a large number of envelope glycoproteins, several of which are predicted to be extensively glycosylated. One such extensively glycosylated envelope proteins is gN, a type I glycoprotein that is particularly interesting for several reasons. It is component of the gM/gN complex which is among the few envelope proteins that are conserved between the herpes viruses indicating an important function of this glycoprotein complex in the biology of herpes viruses [16]. For HCMV, gN is essential for virus replication whereas for alpha-herpes viruses it has been classified as non-essential [17]–[20]. In contrast to alpha-herpesviruses, the gM/gN complex is the most abundant protein complex in the HCMV virion envelope [21]. gN is an extremely polymorphic protein at the amino acid (aa) level. So far four major genotypes have been identified [22]. Differences between the gN genotypes are exclusively located within the surface domain of the protein, reaching amino acid differences of up to 50% between genotypes [22]. The protein is extensively modified by O-linked sugars contributing over 40 kDa of mass to the 15 kDa polypeptide backbone [23]. Despite the enormous amino acid variation in the surface domain of gN, the total number of serine (ser) and threonine (thr) residues remains constant at approximately 50. The conservation of the number of potential glycosylation sites in the face of significant primary sequence variation of non-ser/thr residues in the surface domain of this molecule suggests a strong selective pressure to maintain this precise level of glycan density. In contrast to gN from HCMV, which has a primary sequence of 138 aas, the gN proteins from alpha- and gamma-herpes viruses without exception are smaller molecules of approximately 100 aa that are not predicted to be extensively glycosylated. Experimental data have confirmed the prediction in those cases where the proteins have been studied [18], [24]–[26]. Even within the cytomegalovirus family, gN homologous proteins of the most species are predicted to represent small proteins with limited modifications [27], [28]. The extensive glycosylation of CMV gNs seems to be restricted to viruses derived from the great apes and humans since gN from chimpanzee CMV is predicted to contain a comparable number of O-glycosylation sites to HCMV while gN from rhesus and cynomolgus CMV are short, largely unmodified proteins [29]–[31].

The function(s) of the carbohydrate moieties of gN is largely unknown. The gCII complex, which has been shown to consist of gM and gN, has previously been proposed to be involved in the initial interaction process between the target cells and the virus since it was reported to bind to heparin [32]. Cell-surface heparan sulfate proteoglycans are thought to represent the initial molecules used by the virus to adhere to target cells [33]. With respect to the humoral immune response, gN has been identified as a target of neutralizing antibodies [34]. In fact, in human serum the capacity to neutralize infectious virus *in vitro* is comparable between anti-gN and anti-gB antibodies [34]. Moreover, exchanging the gN-genotypes in a single, genetically homogeneous HCMV strain resulted in strain-specific neutralization by human convalescent sera further emphasizing the importance of gN for the humoral immune response [12].

We hypothesized that the extensive glycosylation of HCMV gN could provide the virus with a mechanism to evade neutralization by antibodies. To test this hypothesis we generated gN-recombinant viruses with reduced carbohydrate modification. Our results indicated that under-glycosylation of gN increased the susceptibility to the neutralizing activity of antibodies directed at gN. Unexpectedly, we also demonstrated that recombinant viruses with under-glycosylated gN were more susceptible to antibodies directed against a number of different virion envelope proteins of HCMV that have been shown to be major targets of the neutralizing antibody response. Together these findings suggest that one function of the extensive glycosylation of gN could be to limit the activity of virus neutralizing antibodies directed at different envelope glycoproteins, a function similar to that of carbohydrates that serve as a glycan shield to limit antibody neutralization of RNA viruses.

## Results

### The formation of the gM/gN complex is not influenced by deletion of amino acid sequences from the surface domain of gN

**Figure 1:**
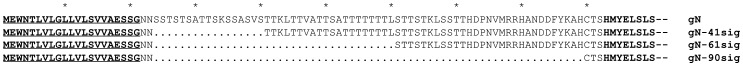
Amino acid sequences of the parent gN protein and the different gN deletions. The amino acid sequence of the gN strain AD169 (residues 1–101) is shown in the top row. Deletions of amino acids are indicated by dots for the truncated molecules. The putative signal sequence (residues 1–21, http://www.cbs.dtu.dk/services/SignalP/) and part of the membrane anchor domain (starting at residue 93, http://www.cbs.dtu.dk/services/TMHMM/) are indicated by bold letters.

More than 50% of the amino acids within the surface domain of gN are ser or thr residues that can serve as substrate for the addition of O-linked sugars. The finding that the viral protein migrates in SDS-PAGE as a 50–60 kDa diffuse species while the theoretical molecular mass of gN is 15 kDa indicating that a significant number of the potential glycosylation sites are utilized [23]. The consequences of this extensive glycosylation to the function of gN and ultimately in the biology of HCMV are unknown. Because site specific mutagenesis of ser and thr residues (total of 36 in HCMV strain AD169) individually and in combination in the surface domain of gN would represent an experiment of considerable complexity, we estimated the impact of reduced glycosylation of gN to the formation of the essential gM/gN complex using mutants constructed by deletion of stretches of ser or thr rich areas of the surface domain of gN. Expression plasmids were constructed that upon transfection into mammalian cells would give rise to truncated gN proteins that could be studied following transient expression. Deletion of aa 24–40 were made to yield plasmid gN-41sig, aa 24–60 and aa 24–89 to yield gN-61sig and gN-90sig, respectively (Figure 1). All plasmids were constructed as to maintain the authentic gN signal sequence which is predicted to be located between aa 1–21. To facilitate protein detection, all proteins were expressed with a myc-epitope at the carboxyl terminus.

**Figure 2:**
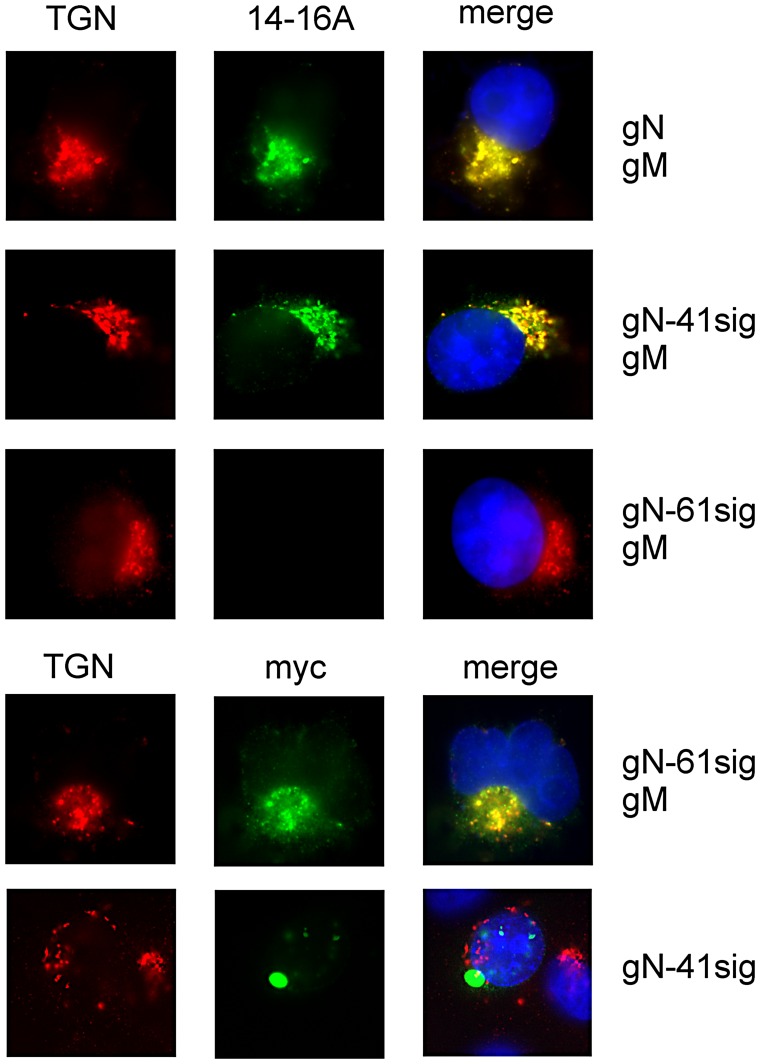
Truncations in gN do not prevent gM/gN complex formation. Cos7 cells were transfected with the indicated plasmids, and protein expression was assayed by reactivity with mab 14-16A (anti-gM/gN), anti-myc (detection of gN) and anti-TGN46 (trans-Golgi apparatus). Reactivity was detected with the appropriate secondary antibodies. The appearance of yellow in the merged pictures indicates colocalization of proteins. In the merge panel cell nuclei are also stained blue.

The plasmids were individually co-transfected with a full length wild type gM encoding plasmid into Cos7 cells and complex formation was analyzed using the gN-specific monoclonal antibody (mab) 14-16A. This antibody has previously been shown to be specific for gN that is complexed with gM [23]. This mab does not react with gN that is not complexed with gM, thus providing an assay for the maintenance of sufficient structure of gN to allow complex formation with gM [23]. Reactivity with mab 14-16A was seen in cells transfected with gM combined with gN and gN-41sig as well as the localization of the protein in the TGN (*trans*-Golgi network), findings that indicated that a complex was formed between these two proteins and that the trafficking of the complex within the cell was similar to the wild type gM/gN complex (Figure 2). No reactivity with mab 14-16A was detected following co-transfection of gM plus gN-61sig, perhaps secondary to a loss of the epitope recognized by mab 14-16A. However, when the cells were stained with an anti-myc antibody we observed reactivity that co-localized the myc-tagged gN with markers for the TGN, indicating formation and correct transport of the gM/gN-61sig complex. Isolated expression of gN results in compact intracellular aggregation of the protein in structures containing endoplasmic reticulum markers and defective transport to the TGN [23]. A similar intracellular traffic defect was observed for the gN-truncated proteins and gN-41sig is shown as example (Figure 2). Transfection using gM combined with the gN-90sig plasmid did not result in a protein that could be detected by immunofluorescence with either antibody suggesting that this deletion resulted in loss of protein structure required for complex formation with gM (data not shown). Together these data demonstrated that deletion of stretches of ser/thr rich sequences of gN could be accomplished without loss of structure required for complex formation with gM and trafficking of this complex to the TGN in transfected cells.

### Recombinant viruses carrying truncated gN replicate normally

**Figure 3:**
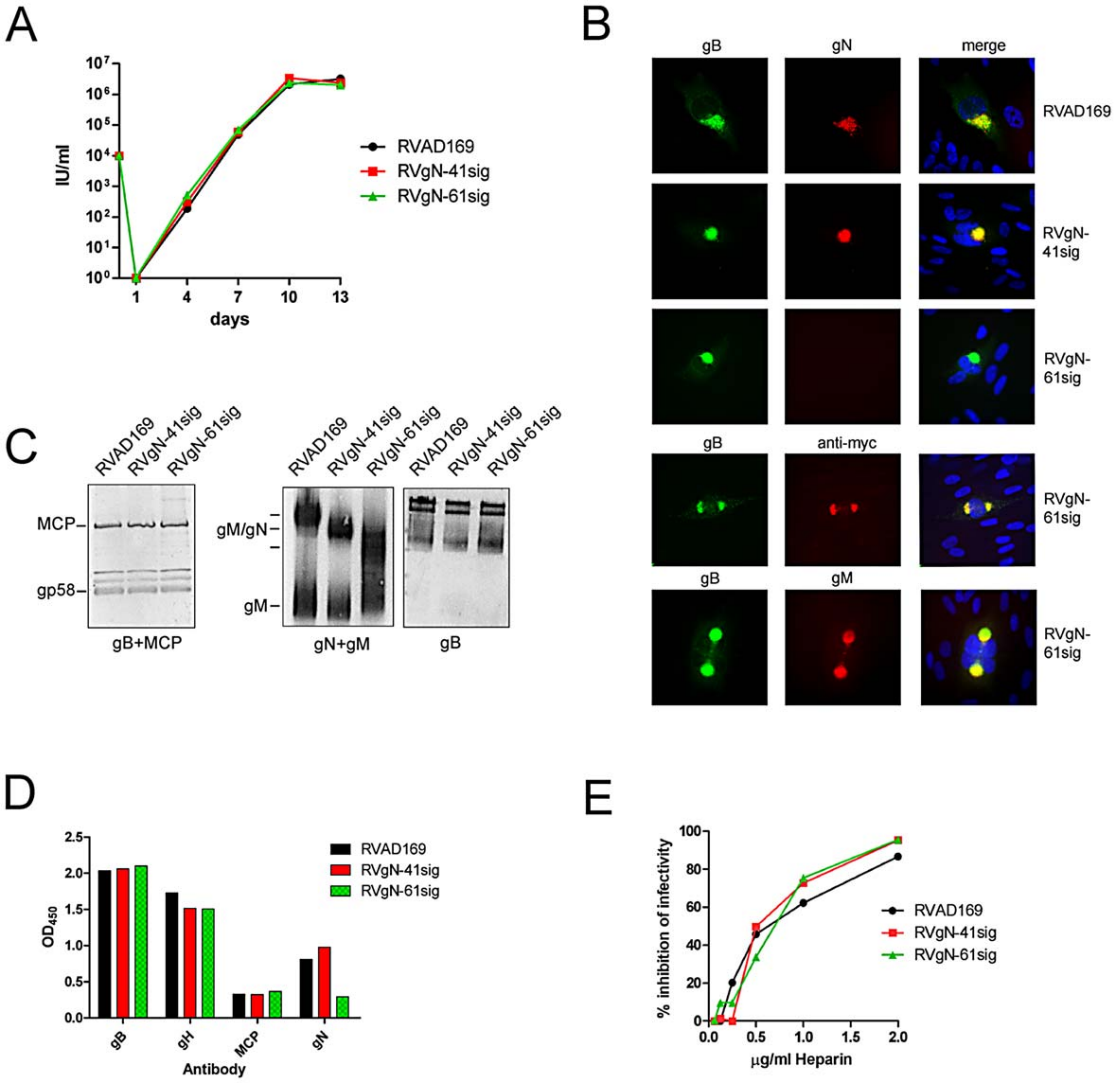
Characterization of the gN-truncated viruses. A) Replication kinetics of gN-truncated viruses. Fibroblasts were infected with identical infectious doses of the different viruses at day zero. At the indicated days post infection supernatants from the infected cultures were harvested and virus titer was determined. B) gM/gN complex formation and intracellular localization in infected fibroblasts. Human fibroblasts were infected with the indicated recombinant viruses and protein expression was assayed 72 h later in indirect immunofluorescence analysis by reactivity with murine mabs specific for gN (14-16A), gM (IMP91-3/1) and gB (human mab C23). The appearance of yellow in the merged pictures indicates colocalization of signals. In the merge panel cell nuclei are also stained blue. C) Analysis of the gM/gN complex in extracellular virus particles. Lysates from gradient purified extracellular virions were subjected to western blot analysis under reducing (left panel) and non-reducing conditions (right panel). The western blot under reducing conditions was developed using antibodies against the major capsid protein (MCP) plus antibody 27–287, recognizing the gp58 part of gB. The analysis under non-reducing conditions was stained using a gM/gN affinity-purified human polyclonal antibody preparation [34] or the gB-specific mab 27–287. D) ELISA analysis of glycoproteins in extracellular virus particles. Plates were coated with lysates from gradient-purified extracellular virions at a concentration of 500 ng/well. Individual proteins were detected by mabs specific for gB (27–287), gH (14-4B), the major capsid protein (MCP), gN (14-16A) and the appropriate secondary antibodies. E) Inhibition of infection by heparin. Heparin was added to extracellular virus for 1 h at the indicated concentrations. The mixture was added to fibroblasts and allowed to infect for 4 h at 37°C. The virus/heparin mixture was removed and percent of infected cells was determined 24 h later by indirect immunofluorescence using an antibody specific for IE-1.

To generate recombinant viruses expressing truncated gN proteins, we followed our previous strategy, i.e. construction of HCMV bacterial artificial chromosomes (BAC) carrying the truncated versions of the gN-gene in place of the full length gene, followed by reconstitution of infectious virus in human cells [19]. All viruses were constructed in the genetic background of HCMV strain AD169 [35]. Replicating virus was recovered for gN versions starting at aa 41 and aa 61 giving rise to RVgN-41sig and RVgN-61sig, respectively. In several attempts no replicating virus could be recovered from BACs carrying the gN-90sig mutation, indicating that this large deletion in gN is lethal, a finding that confirmed our previous results of the essential role of gN for replication of HCMV [36]. RVgN-41sig and RVgN-61sig recombinant viruses replicated with similar efficiency when compared to the parental virus RVAD169, a finding that was consistent with the capacity of these two gN mutants to form a complex with gM and traffic normally in transfected cells. (Figure 3A). In accordance with the similar efficiency of replication of the gN-truncated viruses, we observed no delay in expression of immediate early proteins as determined by indirect fluorescence arguing that the early infection events are similar for the three viruses (data not shown).

To determine if gM/gN complex formation occurred in cells infected with the respective viruses, we carried out indirect immunofluorescence analysis 5 days after infection (Figure 3B). In cells infected with the wild type RVAD169 virus, the gN signal colocalized with gB in a region in close proximity to the nucleus which has been termed the assembly compartment (AC) [37]. In cells infected with RVgN-41sig a similar staining pattern was observed, indicating that the truncated form of gN did not influence the trafficking of the gM/gN complex to the AC. As was demonstrated in transfected cells, expression of the gN-61sig protein could not be detected using the mab 14-16A. However, co-localization of gM and gB in cells infected with RVgN-61sig could be detected and because gM complex formation with gN has been shown to be required for transport of gM from the ER, this finding indicated that the gM/gN-61sig complex had been transported properly to the AC [23]. (Figure 3B). Co-localization of gN-61sig and gB was also observed when gN-61sig was detected via the myc epitope present on gN-61sig (Figure 3B).

To analyze incorporation of the different gM/gN complexes into extracellular virus particles, we gradient purified the respective viruses using ultracentrifugation through glycerol-tartrate gradients and analyzed the viral lysates for the presence of the gM/gN complex by western blot. When virion lysates were analyzed under reducing conditions we did not detect differences in the ratio of the major capsid protein (MCP) and gB between the viruses (Figure 3C).

The gM/gN complex was analyzed under non-reducing conditions since gN migrates as a smear under reducing conditions preventing an accurate estimation of the amount of protein [23]. To detect gN complexes present in all three recombinant viruses we used a gM/gN-specific polyclonal human serum, that was affinity purified from a HCMV hyperimmune globulin preparation [34]. It was shown to be monospecific for gM/gN [34]. The amount of protein that was applied to the analysis was adjusted to give a comparable gB-specific signal for all three viruses (Figure 3C). The results demonstrated that the RVAD169 and RVgN-41sig contained similar amounts of the respective gM/gN complex. For RVgN-61sig, the amount of gM/gN complex was more difficult to estimate due to the diffuse migration of the complex but appeared similar to the other two viruses. The explanation for the diffuse migration of the gM/gN-61sig complex is unknown but a plausible explanation is increased structural heterogeneity of the remaining carbohydrate modifications secondary to loss of a significant number of potential O-linked glycosylation sites. The presence of a similar gM/gN to gB ratio was also confirmed by western blot analyses for RVAD169 and RVgN-41sig using mab 14-16A (data not shown). To obtain more quantitative data on the different proteins in the respective virion particles we performed an ELISA using lysates from gradient purified virions as coating antigen. The ratio of MCP to the envelope proteins gB and gH was comparable for the three different recombinant viruses (Figure 3D). Note that for this analysis an anti-gH mab was used that is dependent on the native conformation of the antigen, indicating that the lysis procedure left the proteins largely intact [38]. Virions from RVgN-61sig gave a reduced signal with mab 14-16A compared to RVAD169 and RVgN-41sig, confirming the results of the indirect fluorescence analysis. Together these data argue that deletion of stretches of ser/thr rich sequences within the surface of domain did not alter the function of the gM/gN complex required for the production of replication competent viruses. Furthermore, the stoichiometry of the three major glycoprotein components of the virion envelope appeared to be unaltered in virions produced by these recombinant viruses suggesting that the deletion of these sequences in the surface domain of gN did not alter the incorporation of the gM/gN complex (or gB, gH) into the envelope of the virus.

The gM/gN complex of HCMV was originally designated gCII complex and it was reported that components of the gCII complex have heparin-binding capacity [32]. Heparin binding is most likely secondary to the carbohydrate modifications of gN since gM is minimally glycosylated and largely buried in the viral envelope. We therefore tested heparin for blocking infection of the gN-truncated viruses. In accordance with previous reports, we observed almost complete inhibition of infection in the presence of 2 µg/ml heparin [39]. Importantly, there was no difference between the three viruses in terms of their capacity to be inhibited by the addition of heparin (Figure 3D). In summary, when combined these data indicated that the behavior of gN-truncated viruses in these *in vitro* assays were phenotypically very similar if not identical to the parental RVAD169.

### Virions carrying truncated gN versions are more susceptible to neutralization by monoclonal antibodies

**Figure 4:**
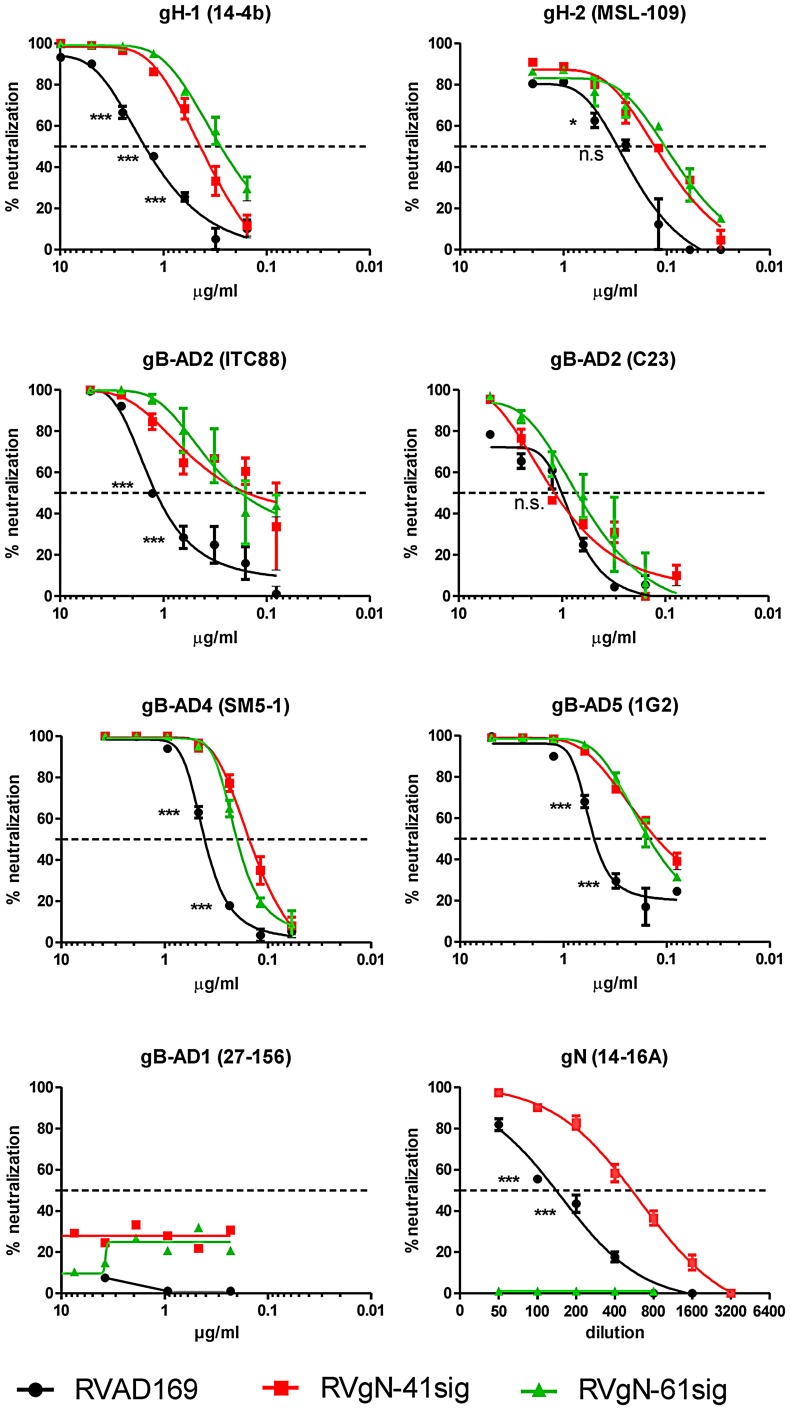
Monoclonal antibodies to gB, gH and gN neutralize the gN-truncated viruses more efficiently than the parent virus. Virus and antibody were incubated for 1 h at 37°C and the mixture was added to HFF monolayers. Cells were washed 4 h later and percent infected cells was determined at 24 h post infection by indirect immunofluorescence using an antibody specific for IE-1. Target proteins and the corresponding mab are indicated. Every mab was tested at least two times with similar results. Data were analyzed by 2way ANOVA and Bonferroni posttests. * p<0.05, **p<0.01, ***p<0.001.

Several possible functions have been suggested as explanations for the extensive carbohydrate modifications of gN including a potential role in cell binding, possibly as a result of its interactions with cell surface glycosaminoglycans and/or serving to limit accessibility of anti-gN antibodies that could neutralize infectious virus. We examined this latter possibility by using the gN truncation mutant viruses in antibody mediated virus neutralization assays. To specifically compare the impact of the loss of carbohydrate modifications and the loss of amino acid sequence on susceptibility of the mutant viruses to neutralization by antibodies, we included antibodies reactive with gN as well as other envelope glycoproteins, gB and gH in these assays. With the exception of 14-16A, an IgM antibody that neutralizes HCMV only in the presence of complement, all antibodies neutralize HCMV in the absence of complement. The gN-specific mab 14-16A showed increased capacity to neutralize RVgN-41sig. RVgN-61sig was not neutralized, which was consistent with the findings that gN-61sig was not recognized by this antibody (Figure 4). These results argued that the loss of carbohydrate and not the antibody recognition site in the RVgN-41sig virus encoded gN was responsible for increased susceptibility to neutralization by this antibody. Moreover, it could also be argued that that carbohydrate modifications on wild type gN functioned to limit the virus neutralizing function of mabs directed against gN. Unexpectedly and perhaps more importantly, both of the gN-truncated viruses were significantly more susceptible to neutralization by other, non-gN specific neutralizing mabs utilized in these assays. The effect was most pronounced for the gH-specific murine mab 14-4b (gH1) and the human anti-gB mab ITC88 (gB-AD2), where differences in 50% neutralization titer of approximately 10-fold were detected. The human anti-gH mab MSL-109 (gH2) and the human anti-gB mabs SM5-1 (gB-AD4), 1G2 (gB-AD5) and C23 (gB-AD2) showed less drastic differences in virus neutralizing activity between the parental and the gN-truncated viruses (Figure 4). Note that the gH-specific mabs and two of the gB-specific mabs (SM5-1, 1G2) depend on native antigen conformation for binding. When non-neutralizing mabs against gB or gH were tested, we found no increase in neutralization activities of these antibodies. The anti-gB mab 27–156 (gB-AD1) is shown as an example. Because the biochemical composition of the envelope as measured by the amounts of three major glycoproteins in the virion envelope was unchanged in the mutant viruses that lacked wild type levels of glycosylation on gN, these results suggested that changes in carbohydrate content of a single envelope glycoprotein were responsible for the increased susceptibility of mutant virions to virus neutralizing antibodies directed at unrelated envelope proteins. These findings raised the possibility that the extensive carbohydrate modifications of gN could be functioning in a similar fashion as the glycan shield that has been proposed for other viruses, including HIV [40], [41].

### The gN-truncated viruses show different susceptibility to neutralization by human sera

**Figure 5:**
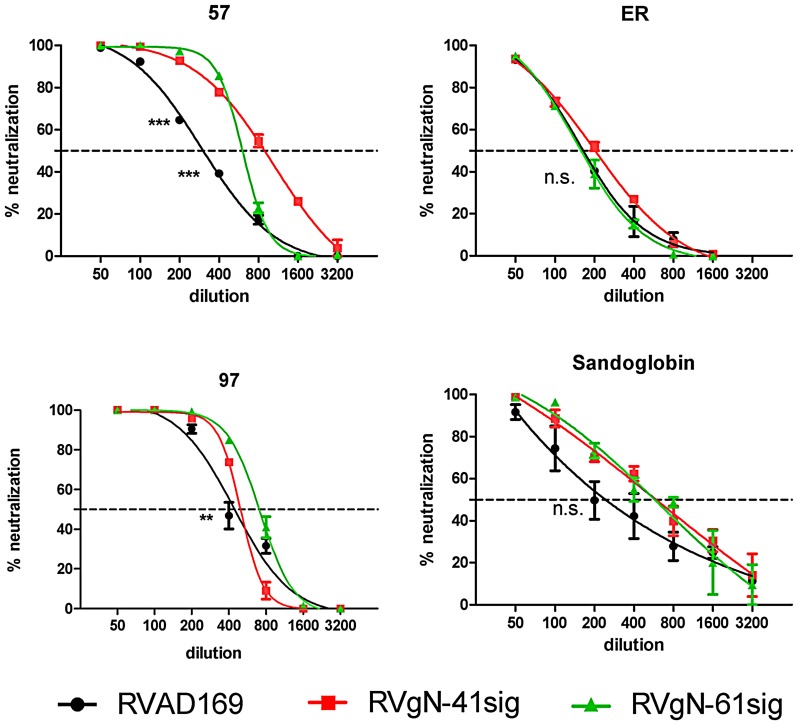
A fraction of polyvalent human sera neutralize the gN-truncated viruses more efficiently than the parent virus. Virus and antibody were incubated for 1 h at 37°C and the mixture was added to HFF monolayers. Cells were washed 4 h later and percent infected cells was determined at 24 h post infection by indirect immunofluorescence using an antibody specific for IE-1. Serum identification is shown above the respective graph. Shown are representative results from at least 3 independent analyses. Data were analyzed by 2way ANOVA and Bonferroni posttests. * p<0.05, **p<0.01, ***p<0.001.

Sera from HCMV infected individuals contain virus neutralizing antibodies directed against a number of different envelope glycoproteins. The majority of antibodies has been suggested to be directed against gB, gH and gN when such sera are analyzed with laboratory strains of HCMV such as strain AD169, which was used in this study [34], [42], [43]. To determine whether the effect observed when mabs were used to neutralize the gN-truncated viruses would be reflected in differences in virus neutralization by polyvalent human sera, we carried out neutralization assays with randomly selected sera from HCMV seropositive donors. A total of 11 specimens were tested and representative results are shown in Figure 5. As could be expected, the polyvalent sera showed less marked differences in neutralization titer between parental virus and the gN-truncated versions than was observed when assays were carried out with single antigen/epitope specific mabs. Two sera showed a significant difference (represented by serum 57 and 97), and the remaining sera differences between 1,3 and 2,2 fold, which, however, did not reach statistical significance (represented by serum ER). In addition, a commercial immunoglobulin preparation (Sandoglobin), presumably derived from a large number of donors, also showed a higher neutralization titer against the viruses expressing the truncated forms of gN, although this difference did not reach significance.

### Mechanistic aspects of neutralization

**Figure 6:**
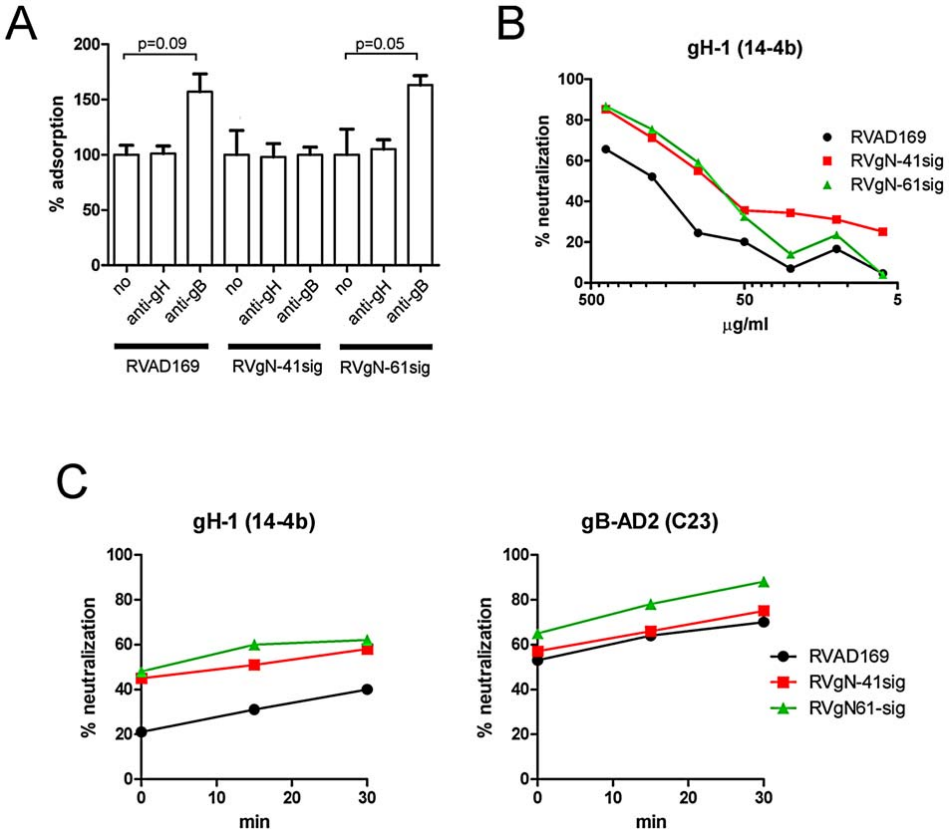
Mechanistic aspects of neutralization. A) Parent virus and gN-truncated viruses adsorb comparably to fibroblasts. Virus (m.o.i. 0.5) was incubated with the indicated antibodies (5 µg/ml) for 1 h at 37°C and cooled to 4°C. The virus/antibody mixture was added to HFF and incubated for 1 h at 4°C. Lysates were prepared and processed for quantitative real time PCR analysis. The virus only sample was set to 100% and used to calculate the remaining samples. B) Post-adsorption neutralization is enhanced for gN-truncated viruses. HFF were adsorbed with virus at a m.o.i. of 0.2 at 4°C for 1 h. Antibody at the indicated concentrations was added and the culture was shifted to 37°C. Extent of infection was analyzed 24 h later by indirect immunofluorescence using an antibody for IE-1. C) gN-truncated viruses are neutralized with faster kinetic than the parent virus. Virus and antibody were mixed at 4°C and added to precooled HFF monolayer cells. The mixture was incubated for the indicated time at 37°C and the percent infected cells was determined at 24 h post infection by indirect immunofluorescence using an antibody specific for IE-1.

We have previously shown that neutralizing anti-gB antibodies do not prevent attachment of fully glycosylated virions to target cells making inhibition of attachment an unlikely mechanism responsible for the increased neutralizing sensitivity of the gN-truncated viruses to anti-gB antibodies [44]. However, the possibility existed that the removal of carbohydrates results in an altered steric orientation of the antibodies bound to the virion surface and thereby provide a new functional property that could alter virion attachment. Therefore, we tested attachment of the different viruses to fibroblast target cells in the presence of antibody. Virus/antibody mixtures were added to target cells at 4°C and the number of HCMV DNA copies attached to the cells was determined by quantitative real time PCR. Attachment of virions to fibroblasts was similar for RVAD169 and the gN-truncated viruses and was not influenced by addition of antibody (Figure 6A). The slight increase of bound virus in the presence of the gB-AD2 specific antibody C23 as compared to control was repeatedly observed and might reflect deposition of antibody/virus aggregates on the surface of cells. Neutralization of HCMV via AD2 specific antibodies requires both arms of the IgG Fab fragment and thus crosslinking of different viruses by C23 is a possibility [45]. We next determined the activity of the mabs towards virus that was adsorbed to cells by pre-adsorbing virus to cells for 1 h at 4°C before the gH-specific mab 14-4b was added. As shown in Figure 6B mab 14-4b was capable of neutralizing HCMV at a postadsorption step. In contrast to our findings from assays of antibody inhibition of attachment, the gN-truncated viruses were more susceptible to neutralization than the parental wild type virus. The higher antibody concentration that was required to completely neutralize adsorbed virus was presumably secondary to the requirement of blocking fusion of an already attached virion.

Finally, to determine whether antibodies that neutralized gN-truncated viruses more efficiently than the parent virus had altered kinetics of virion binding, we performed experiments that allowed an estimate of the rate of antibody-mediated virus neutralization. Virus and antibody were mixed at 4°C and either used immediately to infect cells or warmed to 37°C for 15 min or 30 min before adding to target cells and the percent neutralization was determined 24 h later. As can be seen in Figure 6C, the anti-gH antibody showed increased neutralization capacity towards the gN-truncated viruses at every time point, reflecting the observations that were made in our standard neutralization assays. Neutralization by the gB-AD2 specific human mab C23 was less affected by truncation of gN but clearly detectable (Figure 6C). Again the finding was consistent with the results from the standard neutralization assay as shown in Figure 4 and because the kinetics were similar, argued that the increased susceptibility to virus neutralizing antibody that was seen with the gN truncated viruses was not secondary to alterations in the kinetics of antibody binding to the virion.

In summary, these results indicated that the presence of antibody did not influence the attachment of the gN-truncated viruses to target cells. Moreover, increased neutralization of the gN-truncated viruses was observed very early after combining antibody and virions and even after virions had attached to fibroblasts, suggesting an improved accessibility of target epitopes for the gB- and gH-specific mabs.

### Immunization of mice with the gN-truncated viruses induces antibodies with different specificities and neutralizing activity

**Figure 7:**
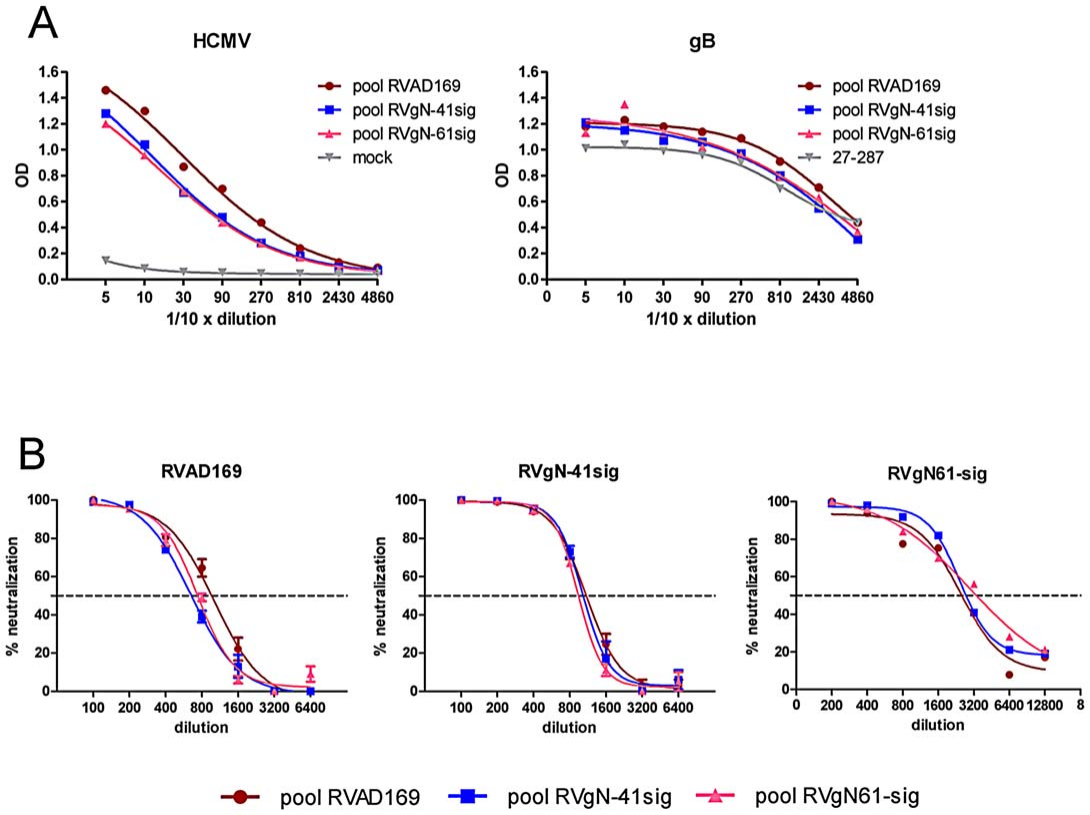
Murine sera raised against the gN-truncated viruses show increased neutralization capacity for the gN-truncated viruses. At an interval of 4 weeks, Balb/c mice were twice immunized i.p. with 5 µg each of gradient purified RVAD169, RVgN-41sig and RVgN-61sig, respectively. Four weeks after the second immunization, animals were boosted with 2 µg virions i.v. and the serum was collected 2 weeks later. Each pool consisted of sera from 3 animals. A) ELISA assay of the pooled sera using gradient purified virus as antigen (left panel) and purified recombinant gB (right panel). Mock: Serum pool from control mice immunized with PBS. 27–287: gB specific mab. B) Neutralization capacity of the serum pools against wild type and the gN-truncated viruses. The serum pools were tested twice with similar results.

The data presented thus far indicated that viruses with truncated gNs differed in the accessibility of epitopes on envelope proteins. Whether these differences would also alter the antigenicity of the viruses was tested in the next series of experiments. Groups of 3 mice each were immunized with equal amounts of gradient purified RVAD169, RVgN-41sig and RVgN-61sig, respectively. The resulting sera from each group were pooled and tested in an ELISA for production of HCMV specific antibodies using RVAD169 as antigen. The individual pools had comparable ELISA titers against whole HCMV antigen as well as against gB alone (Figure 7A). Control mice that had been injected with PBS did not develop anti-HCMV antibodies (Figure 7A). We then tested the serum pools for neutralization of RVAD169 and the gN-truncated viruses (Figure 7B). The individual viruses were neutralized by the different serum pools with similar titers. However, the 50% neutralization titers of the serum pools for RVAD169, RVgN-41sig and RVgN-61sig were different. Whereas, the three serum pools showed 50% neutralization at a dilution of approximately 1∶800 when RVAD169 was used, the titer increased to approximately 1∶3200 when RVgN-61sig was used as target (Figure 7B). 50% neutralization titers for RVgN-41sig by the three serum pools was observed in the range of 1∶1200 (Figure 7B).

**Figure 8:**
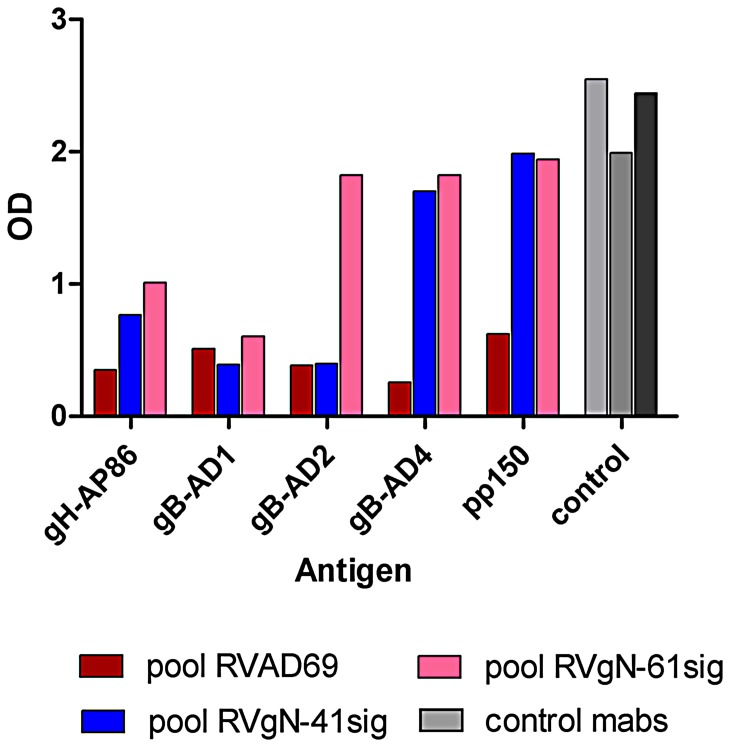
Sera from gN-truncated viruses have different antigen specificity. The individual serum pools were analyzed in an ELISA for reactivity against antigenic domains on gB (AD1, AD2 and AD4), gH (AP86) and the tegument protein pp150 (pp150). Control antibodies for antigen coating of the ELISA plates included 27–287 (gB-AD1, light grey), SA4 (AD86, middle grey) and C23 (gB-AD2, dark grey).

The increased susceptibility of RVgN-61sig to virus neutralizing antibodies raised the question whether this phenomenon was based on an overall increase in neutralizing antibody titer or a specific increase in a selected set of antibodies. To test this, we performed ELISA assays using antigens known for binding of neutralizing antibodies. These included three well characterized antigenic domains on gB, namely AD1, AD2 and AD4 [44], [46], [47]. In addition, the AD86 epitope on gH as well as an antigenic region on the tegument protein pp150 was analyzed [48], [49]. For these analyses, the epitope detected by the anti-gH antibody 14-14b could not be assayed secondary to its conformational dependence. The serum pool derived from RVAD169 immunized mice showed comparable reactivity against epitopes located on gB, gH and pp150 (Figure 8). Sera from mice immunized with the gN-truncated viruses showed a different pattern of reactivity. Whereas reactivity was comparable between all three serum pools for the AD1 epitope on gB and the AD86 epitope on gH, sera from the gN-truncated immunized animals showed drastically enhanced antibody titers against gB AD2, gB AD4 and pp150. Testing the sera individually gave similar reaction pattern indicating that pooling the sera did not bias the result (data not shown). These data suggested that the increase in neutralization capacity in sera from gN-truncated viruses could be based on a selective increase of antibodies binding to neutralization relevant epitopes on different envelope glycoproteins that were less accessible in the wild type AD169 virus.

## Discussion

The remarkable variation in the primary sequence of gN that has been documented in different viral isolates and the extensive oligosaccharide modifications of the gN of HCMV are two characteristics of the HCMV gN that demonstrate its uniqueness among the gNs of human herpes viruses. The observation that the predicted number of potential sites for O-linked carbohydrate modifications in gN remains constant regardless of the variability of the primary sequence argues for a critical role of this modification for the biology of HCMV. Our results are consistent with the possibility that the carbohydrate modifications of this envelope protein limits the activity of virus neutralizing antibodies and suggests at least one functional role for this post-translational modification of this structural protein.

The results of our studies indicated that deletion of ser/thr rich primary sequence of the gN ectodomain and the associated carbohydrate modifications associated with these ser/thr residues had no measurable impact on the formation of the gM/gN complex, localization of the complex in sites of virus assembly, and virus replication. Furthermore, the early events of infection such as adsorption, penetration and expression of immediate-early proteins were similar for both gN-truncated viruses and the wild type parent virus. Likewise, for all viruses in this study attachment was inhibited to a similar extent by the addition of heparin, a finding that argued that either the carbohydrate modifications on gN were unimportant for cell surface proteoglycan binding or that residual carbohydrates on the truncated gNs viruses were sufficient for binding to these cellular molecules. Consistent with the former possibility is the finding that other envelope glycoproteins such as gB can also efficiently bind the heparan sulfate [39]. Overall, our results strongly argued that the deletions introduced into the surface domain of gN did not alter the structure of the molecule so as to limit its functional interactions with gM and its essential role in virus replication. In contrast to these results, deletion of oligosaccharides from gN had a significant impact on the susceptibility to virus neutralization by antibodies suggesting that the oligosaccharides played a role in limiting the activity of virus neutralizing antibodies. The effect was seen for antibodies whose activity depended on binding in *cis*, i.e. gN-specific antibodies. Importantly, deletion of 17 amino acids from the ser/thr sequence in the amino-terminus of gN resulted in a replication competent virus that had increased susceptibility to the neutralizing activity of a mab that reacted with gN from both the wild type and gN truncated virus. Because this antibody recognizes a non-conformation dependent binding site, based on its reactivity with denatured protein, the increased neutralizing activity of this mab for the RVgN-41sig virus is unlikely to be secondary to a conformational change in gN. Together these finding argued that alteration in the carbohydrate modifications of gN in the virus expressing the truncated form of gN likely accounted for the increased virus neutralizing activity of the anti-gN mab when assayed with the RVgN-41sig virus as compared to wild type virus.

Of perhaps greater interest was the finding that deletion of ser/thr rich sequences in the surface domain of gN resulted in viruses that were more susceptible to virus neutralizing activities of antibodies whose binding was in *trans* to gB and gH. This finding was of significance for several reasons. Perhaps the most obvious is that it argued strongly that the mutations introduced into gN that altered the carbohydrate content of gN also had an effect on the recognition of two other abundant envelope glycoproteins by neutralizing antibodies. The mutations in gN did not alter the biochemical content of the envelope as measured by the amount of gN/gM, gB, and gH in the envelope nor the conformation of gB or gH as revealed by their continued recognition by conformation dependent mabs. However, these mutations did alter the susceptibility of these mutant viruses to the neutralizing activity of these mabs. Removal of 17 or 37 residues from the extraviral part of gN resulted in a phenotype that was similar with regard to susceptibility to neutralization. This result indicated that the glycan modification *in toto* and not site specific carbohydrate modification may be required for the inhibition of virus neutralizing antibody activity that is observed *in vitro*. Whether similar requirements are operative *in vivo* is unknown but the maintenance of the number of O-linked glycosylation sites in the coding sequence of gN from a large number of clinical isolates would argue that for optimal evasion of antiviral antibodies, usage of all of the potential carbohydrate modification sites would be required.

Our findings argue for a role for the carbohydrate modifications present on gN in antibody recognition of envelope glycoproteins by virus neutralizing antibodies. The structural relationships between the gM/gN complex, gB, and gH/gL in the AD169 laboratory strain of HCMV are unknown but the complexity of protein composition of the virion envelope would suggest that the structure of the envelope would be complex. Because of this complexity we cannot definitively exclude the possibility that a deletion such as the 17 aa deletion in the amino terminus of the gN41-sig mutant could result in major structural changes in the envelope of the virion once the gM/gN41-sig complex was incorporated. Such structural changes resulting from this mutation could be proposed but not tested with available methodologies. Probing the envelope of the mutant viruses with mabs does argue that the overall structure of the envelope is likely intact and that similar stoichiometric relationships between the three major glycoproteins are maintained, findings that are also consistent with the similar *in vitro* replication of mutant and wild type viruses. However, we cannot formally exclude the possibility that the deletion of residues in the surface domain of gN lead to structural rearrangements within the envelope of infectious virions that increased the neutralizing activity of antibodies directed against three different envelope glycoproteins.

Glycan shields that protect viruses from antibody-mediated neutralization are a well described phenomenon and have been extensively investigated in a number of RNA viruses. The most well studied examples are HIV and influenza virus [40], [41]. For these viruses it is sufficient for the shield to work in *cis* meaning that the protein that carries the sugar is protected by carbohydrate modification, which could be expected since the viruses carry a single multifunctional protein that works in attachment, receptor binding and fusion. Thus, blocking antibody access of a limited set of protein domains that are crucial for the functional activity of neutralizing antibody is sufficient. The envelopes of herpes viruses are considerably more complex carrying several neutralization-relevant targets that exhibit redundant functions in the early events of virus infection. In case of HCMV these include at least gB and different gH complexes [50], [51]. Thus, evasion from neutralizing antibodies would require a potentially very different strategy and one that is likely much more complex because simply protecting a single protein would likely not be sufficient. The induction of neutralizing and non-neutralizing antibodies which compete for binding to the same antigenic domain is a mechanism that has been described for the gB neutralizing site AD1 [13]. Whether such a mechanism is operative for additional antibody binding sites on the HCMV envelope is unknown. More recent data show that there are domains on gB which are bound exclusively by neutralizing antibodies indicating that competition of neutralizing and non-neutralizing antibodies is probably not a general evasion mechanism [44]. Data from the present study raises the possibility that carbohydrate shielding of several glycoproteins by the heavily glycosylaetd gN envelope protein could be a second major mechanism that limits the neutralization of virus infectivity by antibodies. Deletion of a fraction of the predicted oligosaccharide addition sites from gN resulted in increased neutralization activity of a number of mabs that have been shown to be directed at different envelope proteins, although the effect was not equivalent for all antibodies. Whereas one anti-gH antibody and an anti-gB AD2 antibody required 5–10 times higher concentration to achieve 50% neutralization titers towards the gN-truncated viruses, antibodies against other epitopes on gB or gH were less affected by the gN truncations. This finding argues the functional efficiency of the proposed glycan shielding may be heterogeneous depending on the binding activity and target epitope of different mabs. However, with the possible exception of the anti-gB antibody C23, we did not see similar neutralization capacity of any antibody for the parent and the gN-truncated viruses.

We can only speculate on the mechanism(s) that are involved in the glycan mediated shielding of HCMV from the activity of virus neutralizing antibodies. A significant change in the protein composition of the envelope secondary to the gN-truncation could facilitate binding of neutralizing antibodies by drastically altering the structure of the virion envelope. However, this seems unlikely since we did not detect changes in the gB/gN ratio in purified virions nor significant changes in virion binding or internalization. In addition, the observed epitope-specific effect on virus neutralizing antibody activity together with the unchanged stoichiometry of the envelope glycoproteins gB, gH, and gM/gN in recombinant viruses with deletions in the ser/thr rich sequences of gN would argue against a global change in the viral envelope. In support of these arguments, deletion of individual envelope proteins in HSV-1 did not change the composition of others [52]. Thus, it is unlikely that deletion of 17 aa, as in the case of RVgN-41sig, resulted in significant changes in glycoprotein composition or structure of the viral envelope.

An obvious explanation could be the shielding of a fraction of epitopes on gB and gH by the glycans of gN thereby impeding antibody access to selected epitopes. Whether this mechanism is possible given the differences in protein size between gB (approx. 700 aa surface domain), gH (approx. 700 aa surface domain) and gN (approx. 100 aa surface domain) remains to be determined experimentally and will require structural information about the epitopes recognized by neutralizing antibodies that are exposed on the virion surface. Alternatively, removal of sugars from gN could alter the architecture of the outer surface of the envelope without affecting copy numbers of the individual protein components of the envelope. Mass spectrometry of HCMV has identified gM as the most abundant glycoprotein in the viral envelope. It can be assumed that gN is also highly abundant since it is covalently linked to gM via a disulfide bond [21]. The linkage is between cysteine at position 90 in gN and cysteine at position 44 in gM, thus it is not affected by the truncations that were introduced in gN [36] and the western blot analyses of the gN-truncated virions under non-reducing conditions supported this assumption. By occupying space, the bulky carbohydrate head of fully modified gN could result in closer packing of the surface domains of gB and gH, thereby impeding access of some gB- and gH-specific antibodies. If sugar is removed, it may result in a decreased density of the spacing of the respective proteins and permit easier access of antibodies to epitopes on gB and gH. Alternatively, structural changes may be induced resulting in altered binding avidity of immunoglobulin which could affect neutralization sensitivity [53]. The fact that we observed increased susceptibility for only a subset of neutralization relevant epitopes would be compatible with any of these mechanisms.

It is interesting to note, that HCMV carries a number of additional glycoproteins which have been shown to contain significant carbohydrate modifications, such as gB and gO [54]–[56]. If they were to have similar effects, the overall protection from the activity of virus neutralizing antibodies would be very significant. For gB of murid herpes virus 4, a significant effect of glycosylation on the evasion from virus neutralizing antibodies has been demonstrated [57]. There is indirect evidence that gO of HCMV may also protect the virus from neutralizing antibodies. Jiang et al. [58] have reported that the cell-to-cell spread of a recombinant virus lacking gO is more sensitive to neutralization by polyclonal sera than the parental virus. Thus, the protection from antibody-mediated neutralization by glycan modification on envelope glycoproteins could represent a more general phenomenon for herpes viruses than has been previously considered.

The immunization experiments indicated that gN-truncated viruses induced an antibody response that was not different from animals immunized with the parent virus in terms of the overall ELISA titer and gB ELISA titer. However, when the sera were tested in virus neutralization assays, a clear difference was seen. The RVgN-41sig virus and the RVgN-61sig virus were neutralized more efficiently by any serum pool, a result that was most apparent when the RVgN-61sig virus was used in these assays. These findings again emphasize that the gN-truncated viruses were more readily neutralized when compared to the wild type parental virus. Interestingly, on the epitope level, the gN-truncated viruses induced an antibody response that was different from the response against the parent virus. Epitopes which represented minor targets and induced only low levels of antibody in the wild type RVAD169 virus immunized animals became markedly more immunogenic in the gN-truncated virus immunized animals. This was most pronounced for the AD2 and AD4 epitopes on gB but also seen for the epitopes on gH and pp150. Interestingly, this was again an epitope specific effect because this response was not as apparent for the AD1 epitope on gB, possibly because of the dominance of this epitope in the antibody response to HCMV in mice and humans. Thus, the *trans*-effect that was seen in neutralization of the gN-truncated viruses with the different mabs was also reflected after immunization with viruses deficient in the glycan modifications of gN. The underlying mechanisms for the induction of a different set of antibodies by the fully glycosylated virus and the gN-truncated viruses are unclear at present but may involve activation of a different set of naïve B cells to produce antibodies after antigenic stimulation, a mechanism that has recently been suggested for the induction of neutralizing HIV-specific antibodies [59]. Defining the underlying mechanism for the enhanced immunogenicity of selected epitopes on the HCMV envelope proteins is a relevant question for the development of immunogenic components for vaccine development and is actively under investigation in our laboratories. Finally, a recent study of bovine herpes virus 4 that detailed the effect of O-glycosylation of gp180 on antibody evasion also found increased sensitivity of gp180-deficient virions to antibody-mediated neutralization but no difference between the immunogenicity of viruses with or without expression of gp180 [60].

It was surprising that we did not observe a difference in antibody binding ELISA assays when we used whole virus or purified gB as antigenic substrate. The most likely explanation is that the overwhelming composition of the antibody response against either antigen was directed at antibody binding sites irrelevant to the activity of virus neutralizing antibodies. Our analysis of the human antibody repertoire against gB has revealed that >95% of antibodies directed against gB are non-neutralizing [44]. The situation may be similar after immunization of mice with HCMV particles. Increased antibody titers against neutralization relevant epitopes, however, did not translate into increased neutralization capacity of the respective serum pool against the virus carrying full length gN suggesting that cryptic epitopes were not exposed in viruses lacking the full complement of carbohydrate modifications on gN. Thus, the resistance of viruses containing full length fully glyosylated gN can be most readily explained by the shielding by carbohydrates of the respective epitopes recognized by virus neutralizing antibodies. Lastly, what could be the relevance of our findings for the activities of virus neutralizing antibodies *in vivo*? The results from the immunization experiments in mice suggest that individual differences in the antibody repertoire induced in infected individuals in an outbred population such as humans has only limited consequences for the neutralization of the virus *in vivo* since neutralization relevant epitopes might be protected by the glycan shield of the virus.

## Materials and Methods

### Ethics statement

This study was performed in strict accordance with German law (Tierschutzgesetz). The protocol was approved by the Committee on the Ethics of Animal Experiments at the Bavarian Government (Regierung von Mittelfranken, permit 54-2531.31-8/04). All efforts were made to minimize animal suffering.

### Cells and viruses

The human kidney cell line 293T and human lung fibroblasts (MRC5) were maintained in Dulbecco modified Eagle medium supplemented with 10% fetal calf serum (FCS), glutamine (100 mg/L) and gentamycin (350 mg/L). Human foreskin fibroblasts (HFF) were kept in minimum essential medium supplemented with 5% FCS, glutamine and gentamycine. All viruses used were propagated on MRC5 cells and viral titers were determined in HFF using an indirect immunofluorescent assay with the mab p63-27, directed against the HCMV immediate-early 1 (IE1) protein [61]. Virions were isolated by glycerol-tartrate gradient centrifugation as described [62]. For growth curves, HFF, plated in 24-well dishes, were infected at a multiplicity of infection (m.o.i.) of approximately 0.1. After adsorption of virus (2 hours), the inoculum was removed and replaced by fresh medium. Supernatants were harvested at the indicated time points and stored at −80°C until use. Virus titers were determined by an indirect immunfluorescence assay using a mab against the IE-1 protein of HCMV as described [61].

### Antibodies and sera

The mabs used in this study have been described. Murine mab: gM-specific IMP91-3/1 [23], gB-specific 27–287 [63], gN-specific 14-16A [23], gH-specific 14-4b [38]; Human mab: gB-specific C23 (kindly provided by Teijin Pharma Limited, Japan) [64], ITC88 [14], SM5-1 and 1G2 [44]. For specificity of the mabs see Figure S1. Secondary antibodies were purchased from Dianova or DAKO. Sera from HCMV- positive and negative individuals were randomly selected from our diagnostic department.

### Neutralization assay

Serial mab or serum dilutions were incubated with virus preparations for 1 h at 37°C. Viral titers were adjusted to give 100 to 150 infected cells counted on a fluorescence microscope using a 200× magnification, equivalent to 2000 infected cells/15000 cells. The virus antibody mixture was added to fibroblasts which were seeded at 1,5×10^4^ per well in 96-well plates the day before. The medium was replaced 4 h later and the number of infected cells was counted 16 h later using indirect immunofluorescence with mab p63-27. Percent neutralization was calculated as reciprocal of infectivity with maximum infectivity being determined by incubation of virus without antibody. The number of infected cells without addition of antibody also served as reference for the determination of infectious units (IU). For the kinetic experiments, all reagent (virus, antibody, tissue culture medium) were cooled to 4°C before mixing. Mixtures were transferred to 37°C for the indicated time periods and added to fibroblasts at 37°C. The medium was replaced 4 h later and the number of infected cells determined as described above.

### Immunization of mice

Balb/c mice were obtained from Charles River Laboratories, Inc.. Groups of 3 mice each were immunized with the respective virus. 200 µl containing 5 µg of gradient purified virus and 100 µl aluminum hydroxide adjuvant were administered intraperitoneally. Booster immunizations were given at weeks 5 and 8. Animals were sacrificed and sera of mice immunized with the same virus were pooled.

### Plasmids

Plasmids expressing truncated forms of gN were constructed on the basis of pcDNA3.1myc/his (Invitrogen). First, the gN coding sequences for residues 1–23, which includes the predicted signal sequence, was inserted using the EcoRI and BamHI restriction sites of pcDNAmyc/his. In a second cloning step, gN sequences coding for aa 41–138, 61–138 and 90–138 were inserted into the gN-signal sequence containing plasmid via the BamHI and HindIII sites. All DNA fragments were generated by PCR using appropriate primers and integrity of the gN-expressing sequences of the resulting plasmids (gN-41sig, gN-61sig, gN-90sig) was confirmed by nucleotide sequencing.

### BAC mutagenesis and reconstitution of recombinant viruses

Mutagenesis of HB5 [35] was performed using linear DNA fragments for homologous recombination. To generate BACmids containing the respective UL73 mutant sequences we used the plasmid pCPoΔUL73 [19]. This plasmid is a pCPo15 [65] derivative that contains the entire orf UL72 (nt 104560–105730, nomenclature according to Genbank Accession number X17403) at the 5′end and the entire orf UL74 (nt106095–107587) at the 3′end of the kanamycin resistance gene in plasmid pCP-o-15-Link2. Fragments containing the respective UL73 sequences were PCR-amplified from the plasmids described above and the amplimers were inserted into the 5′-flanking region of the kanamycin resistance gene in pCPoΔUL73. From these plasmids PCR fragments were generated encompassing the respective UL73-Kan-UL74 segment. Primers that were used included UL72up5 (nt 105682–105696) and UL73rec3 (nt 106271–106251). Recombination in pHB5 was done as described previously [19]. In brief, the DNA fragment was electroporated into E. coli DH10B carrying the BAC pHB5 and the plasmid pBAD for recE/T mediated recombination [66]. Bacterial colonies were selected on agar plates containing kanamycin (30 µg/ml) and chloramphenicol (30 µg/ml). To confirm the integrity of the recombined BAC, digestion of DNA with the appropriate restriction enzymes was carried out and analyzed via agarose gel electrophoresis in comparison to the parental pHB5. To confirm recombination at the predicted site Southern Blot analysis, PCR analysis as well as DNA sequence analysis of the UL72–UL75 region was performed. To remove the kanamycin resistance gene after successful recombination, plasmid pBT340 encoding the flp-recombinase was used as described [65].

### Reconstitution of the gN-truncated viruses

To reconstitute infectious virus, MRC-5 cells (300.000 cells per well) were seeded into 6-well dishes. 48 h later 5 µg of BAC DNA together with 1 µg of pcDNApp71tag DNA (kindly provided by B. Plachter, University of Mainz, Mainz, Germany) were transfected with Superfect reagent (Qiagen) according to the manufacturer's instructions. 24 hours later culture medium was replaced by fresh medium and cells were cultivated for 7 days. Cells were then transferred to 25 cm^2^ flasks and cultured until a cytopathic effect was observed. The recombinant viruses were designated: RVAD169 (reconstituted from HB5), RVgN-41sig and RVgN-61sig, respectively.

### SDS-PAGE and immunoblotting

Glycerol-tartrate gradient purified virus was subjected to urea-polyacrylamide-gelelectrophoresis as described previously [23]. Transfer of samples to nitrocellulose membranes was carried out by standard procedure. For visualization of antigens, gB-, gN- and gM-specific mabs were applied and detected with peroxidase-conjugated anti-mouse-IgG and anti-mouse-IgM, respectively, and the ECL detection system (Pharmacia Biotech).

### ELISA

The recombinant antigens used for the ELISA have been described [67]. Briefly, the following antigens were used: soluble gB (kindly provided by Sanofi-Pasteur, France); gB-AD1, containing aa 484–650 of gB; gB-AD2, containing aa 68–80 of gB; gB-AD4 containing aa 121–132 and 344–438; gH-AD86 containing aa 1–142 of gH strain AD169 and pp150 containing aa 555–705 of the tegument protein pp150 strain AD169. Proteins were diluted between 25 ng and 200 ng (depending on antigen) in 0.5 M sodium carbonate buffer, pH 9.6, or in 6 M urea (AD1) and 50 µl was used to coat microtiter plates overnight at 4°C. For the ELISA using viral lysates, wells were coated with virus lysates of 500 ng/well in 50 µl in 0.5 M sodium carbonate buffer, pH 9.6. Original virus lysates were prepared at 100 ng/µl in PBS/1% NP40. All subsequent steps were carried out at room temperature. Reaction wells were rinsed with PBS supplemented with 0.1% Tween 20 and blocked for 2 h with PBS containing 2% FCS. Plates were again rinsed with PBS supplemented with 0.1% Tween 20 and incubated with mabs or serum (50 µl/well) for 2 h. Unbound antibody was removed by washing and peroxidase-conjugated anti-human or anti-mouse IgG (Dako, Germany) was added at an appropriate dilution for 1 h. The plate was washed and 100 µl TMB (tetramethylbenzidine) peroxidase substrate, diluted 1∶1 in peroxidase substrate solution B (KPL, USA), added for 5 min. The reaction was stopped by the addition of 100 µl 1 M H_3_PO_4_ and the OD_450_ was determined using an Emax microplate reader (Eurofins MWG Operon, Germany). Dilution of all antibodies was done in PBS with 2% FCS. In all assays involving bacterially derived fusion proteins, the respective prokaryotic fusion partner was assayed in parallel and the optical density was subtracted from values obtained with the fusion protein.

### Virus adsorption and penetration

Fibroblasts were seeded at 3×10^4^ cells per well in 96-well plates. Virus was preincubated with individual mabs for 1 h at 37°C at concentrations ensuring complete neutralization. Cells and the virus/mab mixture were cooled to 4°C and the virus/mab mixture was added to the cells at a multiplicity of infection (m.o.i.) of 0.5. Following incubation for 1 h at 4°C, cells were washed three times with ice-cold PBS and cell lysates were prepared by freezing/thawing. DNA was extracted from the lysates using a MagNA Pure LC (Roche, Germany) instrument and quantitative real-time PCR was performed on an ABI PRISM 7500. To control for recovery of cells, copy numbers of albumine DNA was determined in parallel to HCMV and HCMV copies were calculated per 1000 copies albumine. Primers: CMV 5′:GAGCAGACTCTCAGAGGATCGG; CMV 3′: AAGCGGCCTCTGATAACCAAG; Albumine 5′: GTGAACAGGCGACCATGCT; Albumine 3′: GCATGGAAGGTGAATGTTTCAG.

### Transient expression of gN and image analysis

Cos7 cells grown on glass coverslips in 24-well plates were transfected with 0.8 µg of plasmid DNA using Lipofectamin (Invitrogen). Fibroblasts, also grown on glass coverslips in 24-well plates, were infected with the respective viruses at a m.o.i. of 0.4. At the indicated times, the coverslips were washed and fixed in 3.0% paraformaldehyde in PBS. The fixed cells were permeabilized with PBS, 0.1% Triton X-100 for 4 min and then blocked using PBS 1% BSA for 15 min at room temperature. Primary antibodies were then added for 30 min at 37°C. Following washing, antibody binding was detected with the appropriate secondary antibody conjugated with either FITC or TRITC (Dianova). Images were collected using a Zeiss Axioplan 2 fluorescence microscope fitted with a Visitron Systems CCD camera (Puchheim, Germany). Images were processed using MetaView software and Adobe Photoshop.

## Supporting Information

Figure S1 **Specificity of antibodies in transfected and infected cells.** (A) To demonstrate specificity of antibodies, Cos7 cells were transfected with the indicated plasmids and stained with mabs directed against the myc tag (mab 9E10, Sigma), glycosylated gN (mab 14-16A, Mach et al., 2000, J. Virol. 74: 11881–92) and gM (mab IMP Mach et al., 2000, J. Virol. 74: 11881–92). Binding of primary antibody was detected with the appropriate secondary antibodies (donkey anti-mouse γ-chain specific for anti-myc and IMP; goat anti-mouse μ-chain specific for 14-16A, Dianova). The panels showing the 14-16A staining in the upper row and the myc staining in the middle row were deliberately overexposed to reveal a minimum of background fluorescence. As can be seen, mab 14-16A is not reactive with gN, when transfected in the absence of gM (upper row), the myc-tag specific antibody does not cross-react with gM (middle row) and the mab 14-16A reacts with gN, when cells are cotransfected with a gM-encoding plasmid (lower row). The lower row also demonstrates that gM and gN do colocalize in transfected cells. (B) Fibroblasts were infected with RV-AD69 for 96 h and stained with mabs specific for gB (human mab C23, Meyer et al., 1990, J. Gen.Virol. 71: 2443–50), the myc tag and gM (mab IMP). Binding of primary antibody was detected with the appropriate secondary antibodies (donkey anti-human IgG-specific (Dianova) in case of mab C23). Again, the panel showing the myc staining in the middle row was deliberately overexposed to reveal a minimum of background fluorescence. DAPI staining was used to reveal cell nuclei. None of the antibodies was reactive with non-infected cells and no signal was seen when infected cells were incubated with secondary antibodies alone (data not shown).
(PDF)
