# Supplementary figures and images for: Glycoprotein N of Human Cytomegalovirus Protects the Virus from Neutralizing Antibodies
Source: PLoS Pathog. 2012 Oct 25;8(10):e1002999. doi: 10.1371/journal.ppat.1002999 (PMC3486915; doi:10.1371/journal.ppat.1002999)

**A**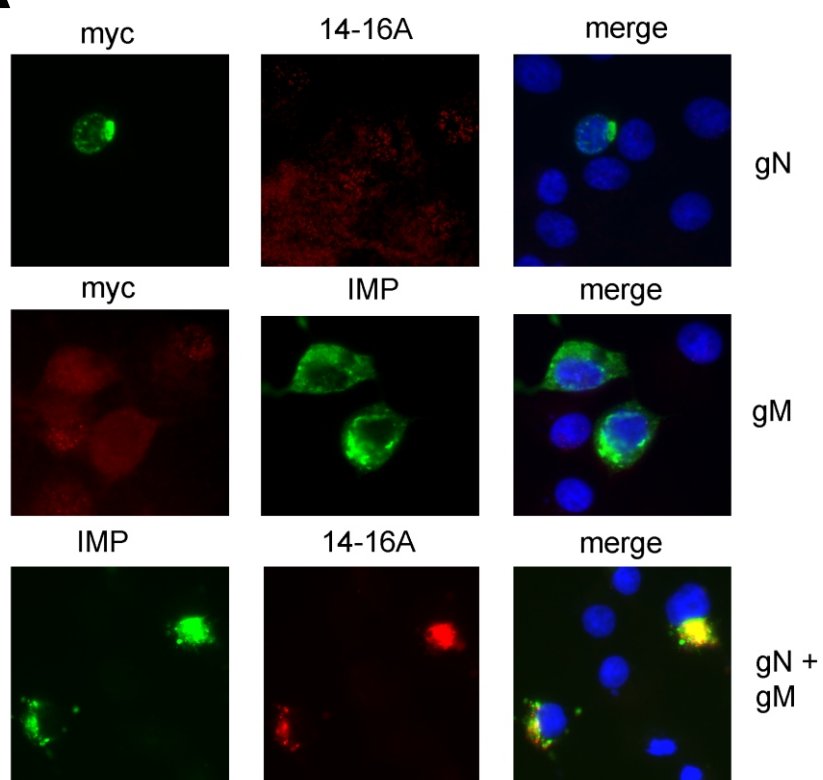**B**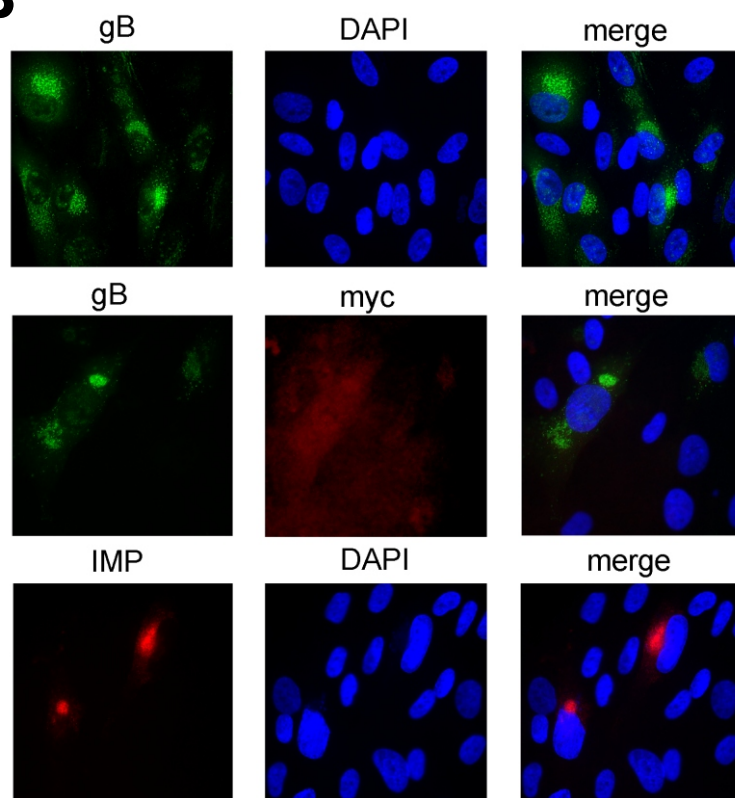

Supplement: Figure S1 — Specificity of antibodies in transfected and infected cells. (A) To demonstrate specificity of antibodies, Cos7 cells were transfected with the indicated plasmids and stained with mabs directed against the myc tag (mab 9E10, Sigma), glycosylated gN (mab 14-16A, Mach et al., 2000, J. Virol. 74: 11881–92) and gM (mab IMP Mach et al., 2000, J. Virol. 74: 11881–92). Binding of primary antibody was detected with the appropriate secondary antibodies (donkey anti-mouse γ-chain specific for anti-myc and IMP; goat anti-mouse μ-chain specific for 14-16A, Dianova). The panels showing the 14-16A staining in the upper row and the myc staining in the middle row were deliberately overexposed to reveal a minimum of background fluorescence. As can be seen, mab 14-16A is not reactive with gN, when transfected in the absence of gM (upper row), the myc-tag specific antibody does not cross-react with gM (middle row) and the mab 14-16A reacts with gN, when cells are cotransfected with a gM-encoding plasmid (lower row). The lower row also demonstrates that gM and gN do colocalize in transfected cells. (B) Fibroblasts were infected with RV-AD69 for 96 h and stained with mabs specific for gB (human mab C23, Meyer et al., 1990, J. Gen.Virol. 71: 2443–50), the myc tag and gM (mab IMP). Binding of primary antibody was detected with the appropriate secondary antibodies (donkey anti-human IgG-specific (Dianova) in case of mab C23). Again, the panel showing the myc staining in the middle row was deliberately overexposed to reveal a minimum of background fluorescence. DAPI staining was used to reveal cell nuclei. None of the antibodies was reactive with non-infected cells and no signal was seen when infected cells were incubated with secondary antibodies alone (data not shown). (PDF) [file ppat.1002999.s001.pdf]
